# Antimicrobial and antibiofilm effects of essential fatty acids against clinically isolated vancomycin-resistant *Enterococcus faecium*

**DOI:** 10.3389/fcimb.2023.1266674

**Published:** 2023-09-29

**Authors:** Ming Wei, Peng Wang, Tianmeng Li, Qiangyi Wang, Mingze Su, Li Gu, Shuai Wang

**Affiliations:** ^1^ Department of Infectious Diseases and Clinical Microbiology, Beijing Institute of Respiratory Medicine and Beijing Chao-Yang Hospital, Capital Medical University, Beijing, China; ^2^ Department of Clinical Laboratory, Beijing Hospital, National Center of Gerontology, Institute of Geriatric Medicine, Chinese Academy of Medical Sciences, Beijing, China; ^3^ Department of Clinical Laboratory, Beijing Tongren Hospital, Capital Medical University, Beijing, China

**Keywords:** enterococcus faecium, multidrug resistance, biofilm, VRE, fatty acid

## Abstract

**Introduction:**

*Enterococcus faecium* is a leading cause of hospital-acquired infections, which has become a serious public health concern. The increasing incidence of vancomycin-resistant *E. faecium* (VRE-fm) raises an urgent need to find new antimicrobial agents as a complement to traditional antibiotics. The study aimed to evaluate the antimicrobial and antibiofilm activity of essential fatty acids (EFAs) against VRE-fm, and further explore the molecular mechanism of the antibiofilm activity of EFAs.

**Method:**

The microdilution broth method was used for antimicrobial susceptibility testing with traditional antibiotics and EFAs, including α-linolenic acid (ALA), eicosapentaenoic acid (EPA), docosahexaenoic acid (DHA), linoleic acid (LOA), γ-linolenic acid (GLA), and arachidonic acid (AA). The effect of EFAs on cell morphology of VRE-fm was investigated by scanning electron microscopy. The crystal violet method was used to evaluate the antibiofilm activities of EFAs against VRE-fm. Furthermore, the expression of biofilm-related genes (*acm*, *atlA*, *esp*, and *sagA*) of VRE-fm isolates under the action of GLA was analyzed using quantitative reverse transcription PCR (qRT-PCR) assay.

**Results:**

VRE-fm isolates were highly resistant to most traditional antibiotics, only highly susceptible to quinupristin-dalfopristin (90.0%), tigecycline (100%), and linezolid (100%). EPA, DHA, and GLA exhibited excellent antimicrobial activity. The MIC_50/90_ of EPA, DHA, and GLA were 0.5/1, 0.25/0.5, and 0.5/1 mM, respectively. SEM imaging showed that strain V27 adsorbed a large number of DHA molecules. Furthermore, all EFAs exhibited excellent inhibition and eradication activities against VRE-fm biofilms. The biofilm inhibition rates of EFAs ranged from 45.3% to 58.0%, and eradication rates ranged from 54.1% to 63.4%, against 6 VRE-fm isolates with moderate biofilm formation ability. GLA exhibited remarkable antibiofilm activity against VRE-fm isolates. The qRT-PCR analysis showed that GLA could significantly down-regulate the expression of the *atlA* gene (*P* < 0.01) of VRE-fm.

**Conclusion:**

DHA showed the strongest antibacterial activity, while GLA showed the strongest antibiofilm effect among the EFAs with antibacterial activity. Our novel findings indicate that the antibiofilm activity of GLA may be through down-regulating the *atlA* gene expression in VRE-fm. Therefore, DHA and GLA had the potential to be developed as therapeutic agents to treat infections related to multiple antimicrobial-resistant *E. faecium*.

## 1 Introduction

*Enterococcus faecium* (*E. faecium*) is a Gram-positive member of the gut microbiota in humans. Commensal intestinal *E. faecium* is not pathogenic in healthy hosts but can cause infection in susceptible ones (Ch’ng et al., 2019). *E. faecium* is a leading cause of hospital-acquired infections that are often difficult to treat due to multiple antimicrobial resistances (Santajit and Indrawattana, 2016; Panda et al., 2022). In recent years, new variants of *E. faecium* have emerged that are better adapted to the healthcare environment, which are prone to outbreaks of infection (O’Toole et al., 2023). Infections caused by vancomycin-resistant *E. faecium* (VRE-fm) have further reduced treatment options due to increased resistance to a wide range of antibiotics (Lagatolla et al., 2022; Cairns et al., 2023). In addition, the ability of *E. faecium* to form biofilms is its remarkable pathogenicity characteristic, and the biofilm greatly enhances antibiotic resistance compared to planktonic cells (Ch’ng et al., 2019; Patil et al., 2021). Therefore, the infection caused by VRE-fm is a serious challenge worldwide, and it is urgent to develop new antimicrobial agents to combat VRE-fm, as well as to inhibit and eradicate the biofilm formed by VRE-fm.

Unsaturated fatty acids are well known to display antimicrobial activity (Desbois, 2012; Yoon et al., 2018). However, due to the discovery and development of traditional antibiotics, research on the antimicrobial properties of unsaturated fatty acids has been neglected for a long time. With the increasing severity of traditional antibiotic resistance, unsaturated fatty acids have been reconsidered as antimicrobial agents. Recently, numerous studies indicated that unsaturated fatty acids could also inhibit or eradicate biofilms formed by various microbial pathogens (Kim et al., 2018; Ramanathan et al., 2018; Cui et al., 2019; Wang et al., 2022). Consequently, unsaturated fatty acids could represent the next generation of antimicrobial agents to treat and prevent biofilm-associated infections.

Essential fatty acids (EFAs) that the body needs but cannot make and must get from food, belong to unsaturated fatty acids and play an important role in the body. EFAs are based on α-linolenic acid (ω-3 group) and linoleic acid (ω-6 group), and correlated with infant development, reduction of cardiovascular morbidity and mortality, cancer prevention, brain and vision functioning, etc. (Kaur et al., 2014) EFAs are not only nontoxic, but also essential nutrients for the human body. Therefore, it is a good model to carry out research on antimicrobial and antibiofilm using EFAs as representative of novel antimicrobial agents.

In the context, three ω-3 group including α-linolenic acid (ALA, 18:3), eicosapentaenoic acid (EPA, 20:5), and docosahexaenoic acid (DHA, 22:6), and three ω-6 group members including linoleic acid (LOA, 18:2), γ-linolenic acid (GLA; 18:3) and arachidonic acid (AA, 20:4) were used to evaluate the antimicrobial and antibiofilm effects against VRE-fm *in vitro*. In addition, for the first time, we study the antibiofilm mechanism of GLA against VRE-fm. Our study indicates that GLA may be developed as a novel therapeutic agent to treat VRE-fm and biofilm-related infections.

## 2 Results

### 2.1 Antibiotic resistance profile of VRE-fm

**Figure 1:**
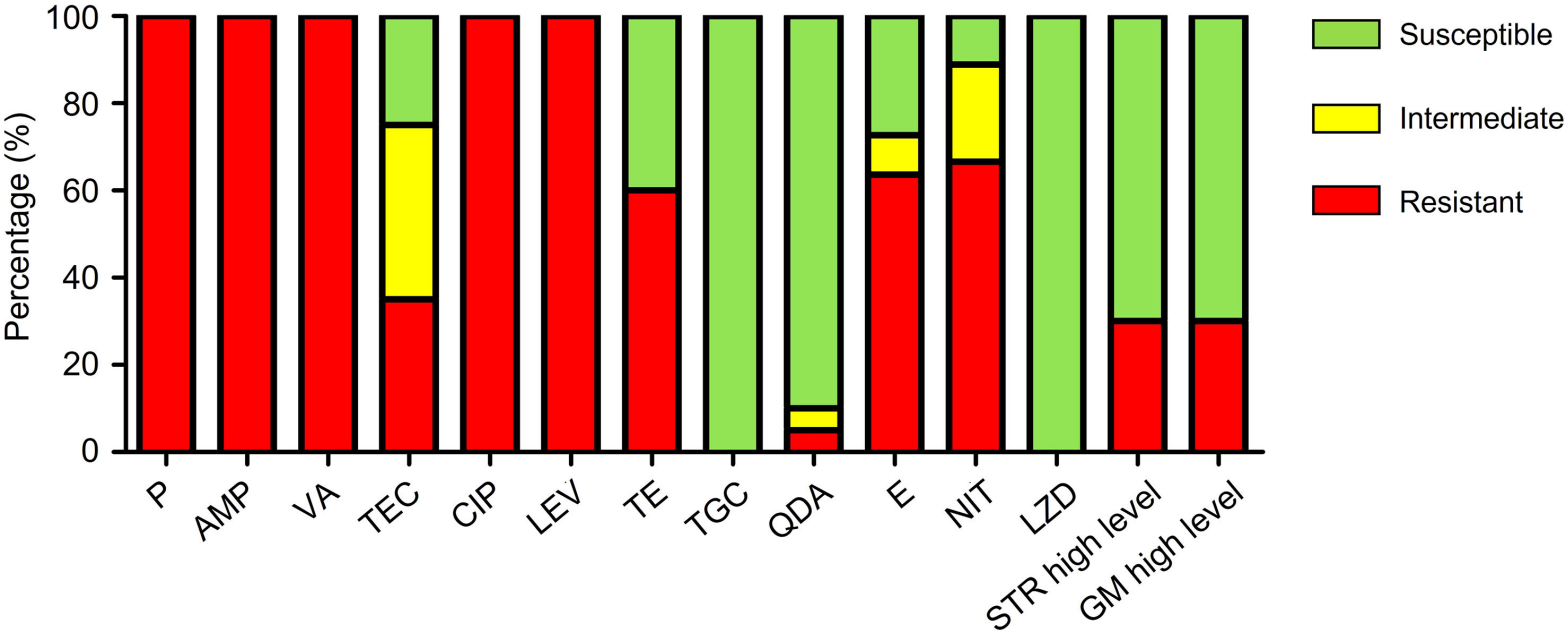
Antibiotic resistance patterns of VRE-fm isolates (n = 20) to 14 antibiotics. P, penicillin; AMP, ampicillin; VA, vancomycin; TEC, teicoplanin; CIP, ciprofloxacin; LEV, levofloxacin; TE, tetracycline; TGC, tigecycline; QDA, quinupristin-dalfopristin; E, erythromycin; NIT, nitrofurantoin; LZD, linezolid; STR, streptomycin; GM, gentamicin.

The antibiotic resistance profile of 20 isolates of VRE-fm is shown in **Table 1** and summarized in **Figure 1**. The VRE-fm isolates in the study were highly resistant to penicillin (100%), ampicillin (100%), ciprofloxacin (100%), levofloxacin (100%), nitrofurantoin (66.7%), erythromycin (63.6%), and tetracycline (60.0%), and were moderately resistant to teicoplanin (35.0%), high-level streptomycin (30.0%), and high-level gentamicin (30.0%), while they were only highly susceptible to quinupristin-dalfopristin (90.0%), tigecycline (100%), and linezolid (100%).

**Table 1:** Antimicrobial susceptibility of VRE-fm isolates.

Strains	Source	P	AM	VA	TEC	CIP	LEV	TE	TGC	QDA	E	NIT	LZD	high-level STR	high-level GM
V03	Drain fluid	R	R	R	I	R	R	S	S	S	S	–	S	S	S
V05	Drain fluid	R	R	R	R	R	R	R	S	S	S	–	S	R	S
V09	Drain fluid	R	R	R	I	R	R	R	S	S	R	–	S	S	S
V10	Drain fluid	R	R	R	S	R	R	R	S	S	R	–	S	S	S
V16	Drain fluid	R	R	R	I	R	R	R	S	S	R	–	S	S	R
V18	Drain fluid	R	R	R	R	R	R	S	S	S	I	–	S	S	S
V23	Drain fluid	R	R	R	R	R	R	S	S	S	S	–	S	S	S
V27	Drain fluid	R	R	R	I	R	R	R	S	S	R	–	S	S	R
V29	Drain fluid	R	R	R	S	R	R	R	S	S	R	–	S	S	R
V32	Drain fluid	R	R	R	I	R	R	S	S	S	R	–	S	S	S
V33	Drain fluid	R	R	R	I	R	R	S	S	S	R	–	S	R	S
V06	Urine	R	R	R	R	R	R	R	S	S	–	R	S	R	S
V08	Urine	R	R	R	S	R	R	S	S	S	–	R	S	R	S
V14	Urine	R	R	R	S	R	R	R	S	S	–	I	S	S	S
V19	Urine	R	R	R	I	R	R	R	S	S	–	I	S	S	S
V20	Urine	R	R	R	S	R	R	R	S	R	–	R	S	R	S
V21	Urine	R	R	R	R	R	R	R	S	I	–	R	S	S	R
V22	Urine	R	R	R	R	R	R	S	S	S	–	R	S	R	R
V26	Urine	R	R	R	R	R	R	R	S	S	–	R	S	S	R
V30	Urine	R	R	R	I	R	R	S	S	S	–	S	S	S	S

S, sensitive; I, intermediate sensitive; R, resistant; P, penicillin; AMP, ampicillin; VA, vancomycin; TEC, teicoplanin; CIP, ciprofloxacin; LEV, levofloxacin; TE, tetracycline; TGC, tigecycline; QDA, quinupristin-dalfopristin; E, erythromycin; NIT, nitrofurantoin; LZD, linezolid; STR, streptomycin; GM, gentamicin.

“-”, untested.

### 2.2 Antimicrobial activity of EFAs on planktonic VRE-fm

The MIC values of different EFAs against VRE-fm isolates in the planktonic form are listed in **Table 2**. Within the set range of EFA concentrations, the results showed that LOA and AA did not have antibacterial activity. By comparing MIC_50_ and MIC_90_, it was found that DHA exhibited the best antimicrobial activity, while EPA and GLA showed slightly lower than DHA, and ALA had a weak inhibitory effect on VRE-fm isolates.

**Table 2:** The MICs of EFAs against VRE-fm isolates.

Isolates	MIC (mM) *^a^*					
	ALA	EPA	DHA	LOA	GLA	AA
V03	1	0.5	0.25	>1	0.5	>1
V05	>1	0.5	0.25	>1	0.5	>1
V09	>1	0.5	0.25	>1	1	>1
V10	1	1	0.25	>1	1	>1
V16	>1	1	0.5	>1	1	>1
V18	>1	0.5	0.25	>1	1	>1
V23	>1	0.5	0.25	>1	0.5	>1
V27	1	0.25	≤0.125	>1	0.5	>1
V29	1	0.5	≤0.125	>1	0.25	>1
V32	1	0.25	≤0.125	>1	0.5	>1
V33	1	0.5	0.5	>1	1	>1
V06	>1	0.5	0.25	>1	1	>1
V08	1	0.5	0.25	>1	0.5	>1
V14	>1	0.5	0.25	>1	0.5	>1
V19	1	0.5	0.25	>1	0.5	>1
V20	>1	0.5	0.5	>1	1	>1
V21	>1	0.5	0.5	>1	1	>1
V22	>1	1	0.5	>1	1	>1
V26	0.5	0.25	≤0.125	>1	0.25	>1
V30	0.5	0.25	≤0.125	>1	0.25	>1
MIC_50_ *^b^*	1	0.5	0.25	>1	0.5	>1
MIC_90_ *^b^*	>1	1	0.5	>1	1	>1

^*a*^The MIC was defined as the lowest concentration at which there is a 100% reduction in the growth control (absence of drug).

^*b*^MIC_50/90_, MIC for 50% and 90% of the isolates, respectively.

MIC, minimum inhibitory concentration; EFA, essential fatty acid; ALA, α-linolenic acid; EPA, eicosapentaenoic acid; DHA, docosahexaenoic acid; LOA, linoleic acid; GLA, γ-linolenic acid; AA: arachidonic acid.

### 2.3 Morphological changes observed by scanning electron microscope

**Figure 2:**
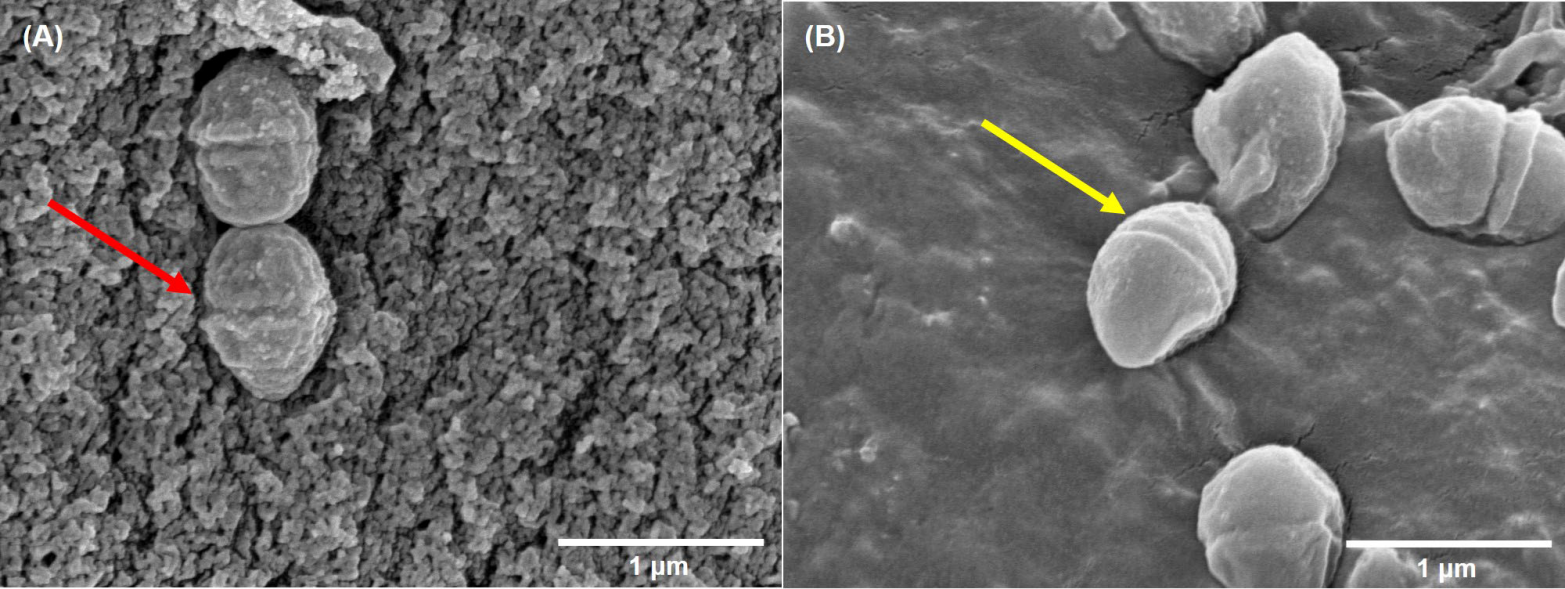
The scanning electron microscope imaging. **(A)** incubated with DHA; **(B)** incubated without DHA. The red arrow indicates rough cells; The yellow arrow indicates smooth cells.

Compared to control group, a large number of DHA molecules were adsorbed to the cell wall of strain V27 in the experimental group, and the surface became rough (**Figure 2**). When observing a large number of fields, bacterial cells with significantly damaged cell membranes were not observed.

### 2.4 Biofilm formation ability of VRE-fm

**Figure 3:**
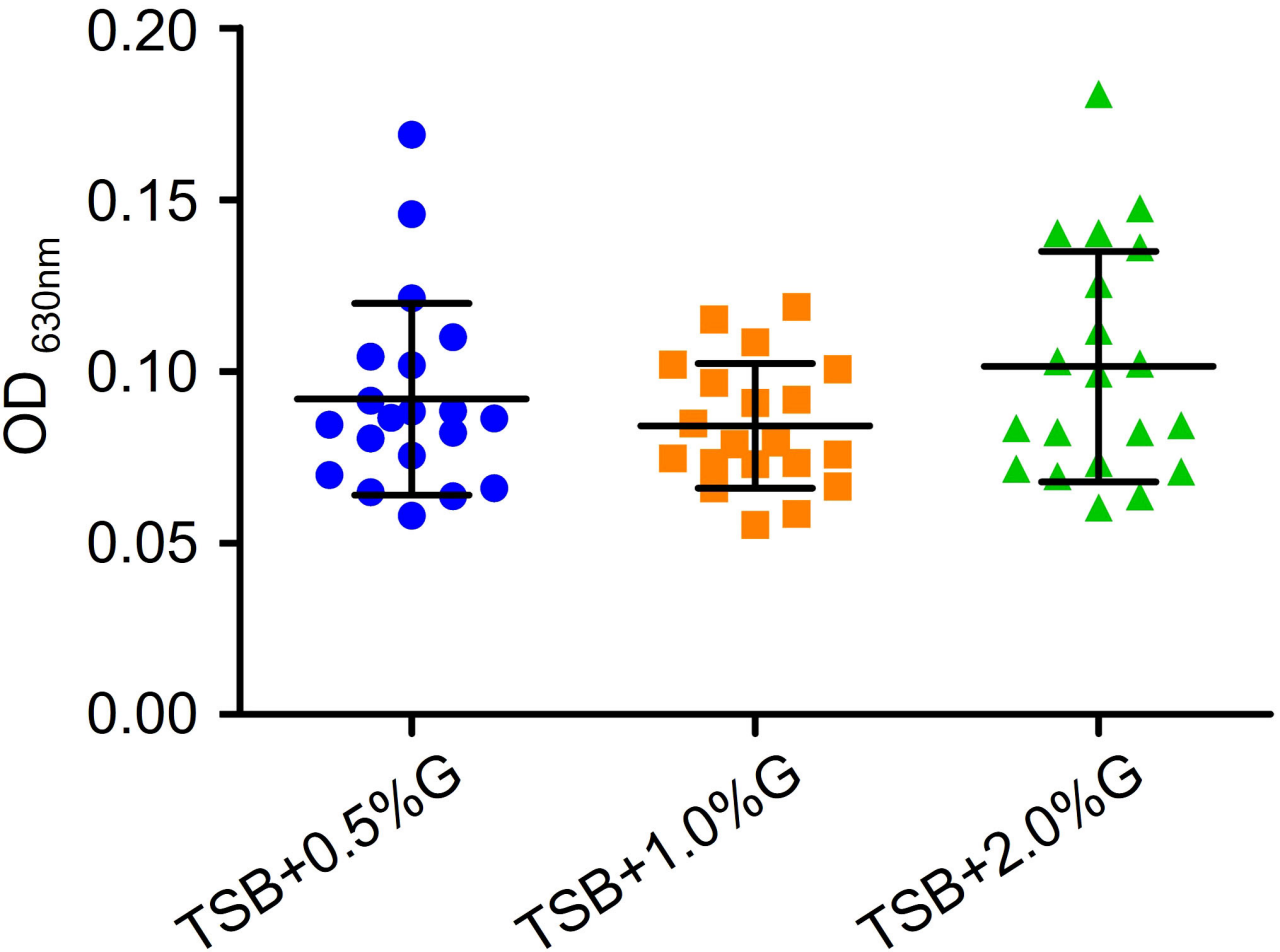
Comparison of the effects of different concentrations of glucose in TSB broth on the biofilm formation of VRE-fm isolates. G, glucose.

To investigate the antibiofilm effect of EFAs, we explored the optimal condition for biofilm formation *in vitro*. Different concentrations of glucose were added to trypticase-soy broth (TSB) to test the biofilm formation of VRE-fm isolates. The results are shown in **Figure 3**. Although there was no statistically significant difference in biofilm biomass when different concentrations of glucose were added, the mean value of biofilm biomass in the TSB+2% glucose group was the highest. Therefore, TSB containing 2% glucose (TSBG) medium was chosen as the final condition in the study.

**Figure 4:**
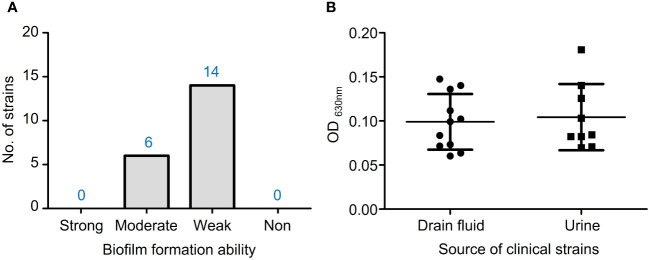
**(A)** The biofilm formation ability of VRE-fm isolates in TSB+2% glucose; **(B)** Comparison of the biofilm biomass of VRE-fm isolates from different clinical sources.

In the TSBG medium, 6 out of 20 isolates had moderate biofilm formation ability, while 14 isolates had weak ability (**Figure 4A**). There were no statistically significant differences in biofilm formation capacity between VRE-fm isolates from drain fluid and urine sources (**Figure 4B**).

### 2.5 Antibiofilm effect of EFAs

**Figure 5:**
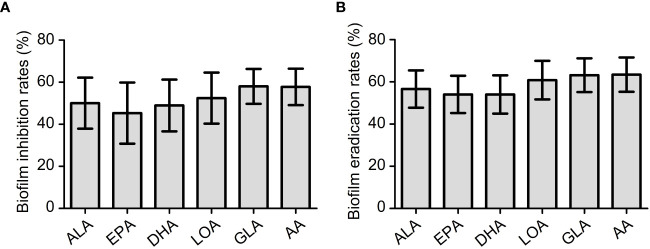
**(A)** The biofilm inhibition rates (%) of EFAs against VRE-fm isolates; **(B)** The biofilm eradication rates (%) of EFAs against VRE-fm isolates. ALA, α-linolenic acid; EPA, eicosapentaenoic acid; DHA, docosahexaenoic acid; LOA, linoleic acid; GLA, γ-linolenic acid; AA, arachidonic acid.

To study the antibiofilm activity of these EFAs, 6 isolates of VRE-fm (V05, V06, V08, V09, V22, and V27) with moderate biofilm formation ability were selected for the experiments. The antibiofilm activities of the EFAs against the VRE-fm isolates were different from those of the antimicrobial activities. All EFAs at 1 mM exhibited excellent activity in inhibiting and eradicating biofilms (**Figure 5**). The biofilm inhibition rates for EPA, DHA, ALA, LOA, AA, and GLA against these VRE-fm isolates were 45.3%, 48.9%, 50.0%, 52.4%, 57.8%, and 58.0%, respectively (**Figure 5A**). While the biofilm eradication rates for EPA, DHA, ALA, LOA, GLA, and AA were 54.1%, 54.1%, 56.6%, 60.8%, 63.2%, and 63.4%, respectively (**Figure 5B**). GLA showed the best antibiofilm activity against VRE-fm among the EFAs with antibacterial activity.

### 2.6 Detection of biofilm-related genes in VRE-fm

In all VRE-fm isolates, the genes *acm*, *esp*, and *sagA* were detected in 20/20 (100%). Only one isolate with low biofilm forming capacity did not detect the *atlA* gene (95.0%, 19/20). However, *agg* and *gelE* genes were not detected in any isolates (**Table 3**).

**Table 3:** PCR detection of biofilm related genes in VRE-fm isolates.

Ioslates	Biofilm-related genes					
	*acm*	*agg*	*atlA*	*esp*	*gelE*	*sagA*
V03	+	–	+	+	–	+
V05	+	–	+	+	–	+
V06	+	–	+	+	–	+
V08	+	–	+	+	–	+
V09	+	–	+	+	–	+
V10	+	–	+	+	–	+
V14	+	–	+	+	–	+
V16	+	–	+	+	–	+
V18	+	–	+	+	–	+
V19	+	–	+	+	–	+
V20	+	–	+	+	–	+
V21	+	–	+	+	–	+
V22	+	–	+	+	–	+
V23	+	–	+	+	–	+
V26	+	–	+	+	–	+
V27	+	–	+	+	–	+
V29	+	–	+	+	–	+
V31	+	–	+	+	–	+
V32	+	–	–	+	–	+
V33	+	–	+	+	–	+

“+”, detected; “-”, undetected.

### 2.7 Modulation of biofilm-related genes expression in VRE-fm by GLA

**Figure 6:**
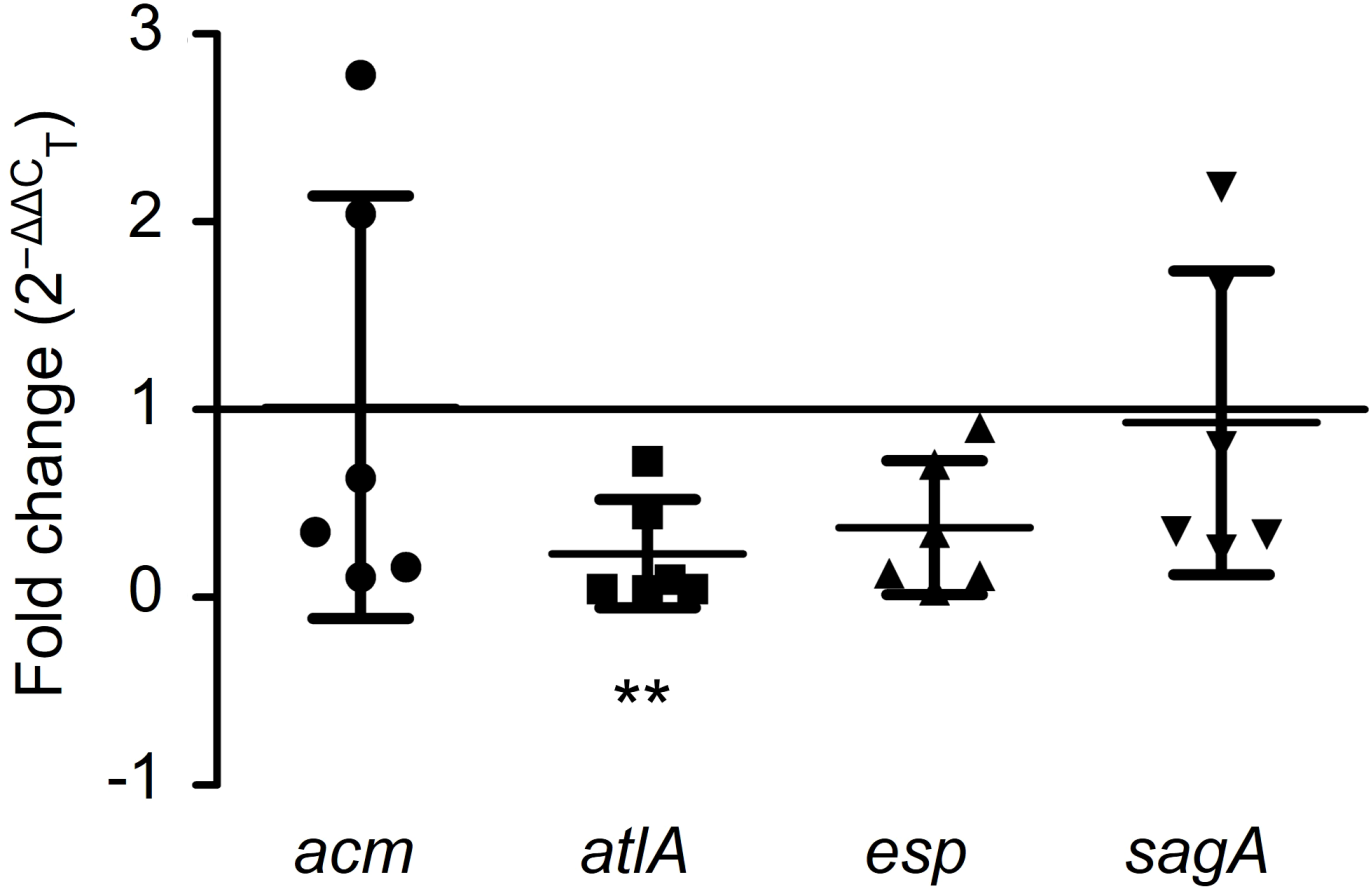
The expression levels (fold change) of biofilm-related genes in mature biofilms of the six VRE-fm isolates treated with γ-linolenic acid (GLA). **P < 0.01 compared to control by Dunnett’s t test.

To illustrate the antibiofilm mechanism of GLA, the expression levels of the biofilm-related gene (*acm*, *atlA*, *esp*, and *sagA*) in six VRE-fm isolates were analyzed. Compared to the control group, GLA down-regulated the expression of the VRE-fm *atlA* gene (0.232-fold) and the difference was statistically significant (*P* < 0.01), while the gene expression levels of *acm* (1.013-fold), *esp* (0.373-fold), and *sagA* (0.931-fold) compared to the control had no statistical difference (*P* > 0.05) (**Figure 6**).

**Figure 7:**
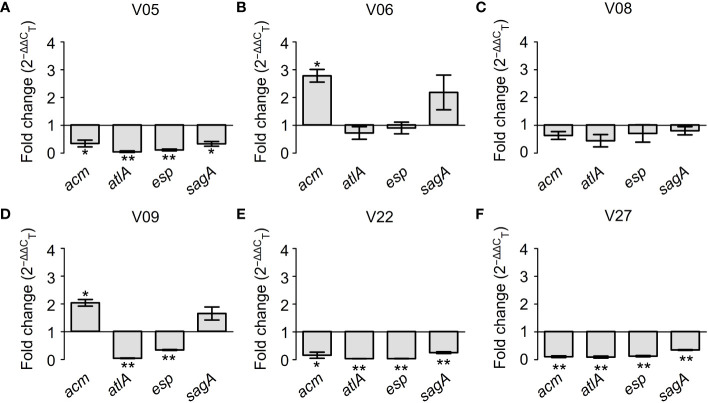
The expression levels (fold change) of biofilm-related genes in mature biofilms of each VRE-fm isolate treated with γ-linolenic acid (GLA). *P < 0.05 and **P < 0.01 compared to the control by the Dunnett’s *t* test. **(A)** V05; **(B)** V06; **(C)** V08; **(D)** V09; **(E)** V22; **(F)** V27.

**Figure 7** further demonstrated the regulatory effects of GLA on the expression levels of biofilm-related genes in different VRE-fm isolates. Among six tested VRE-fm isolates, the expression levels of the *acm*, *atlA*, *esp*, and *sagA* genes were down-regulated in four isolates (V05, V08, V22 and V27). Compared to the control group, there was a significant difference in the GLA-treated groups of V05, V22 and V27 but there was no significant difference for V08. Meanwhile, compared to other isolates, the expression levels of *acm* and *sagA* genes were different in the treatment groups of V06 and V09 isolates, which were up-regulated in GLA-treated groups.

## 3 Discussion

The emergence of VRE-fm poses a significant threat to public health. Additionally, the ability of *E. faecium* to form biofilms further enhances its resilience and makes it challenging to eradicate infections caused by this pathogen. In recent years, there has been growing interest in the potential antimicrobial and antibiofilm effects of EFAs against clinically isolated VRE-fm. In this study, we not only evaluated the antimicrobial and antibiofilm abilities of EFAs against VRE-fm but also first analyzed the possible molecular mechanisms of GLA eradication of VRE-fm biofilms.

In this study, the VRE-fm isolates were highly resistant to most traditional antibiotics, only highly susceptible to quinupristin-dalfopristin (90.0%), tigecycline (100%), and linezolid (100%). Therefore, infections caused by VRE-fm are difficult to treat due to the limited choice of antibiotics. The intrinsic resistance to β-lactams (penicillin and ampicillin) of VRE-fm strains is due to the high expression of penicillin-binding proteins that have low affinity to β-lactams, allowing peptidoglycan synthesis and bacterial growth (Montealegre et al., 2017), which makes enterococci about 100 times more resistant than streptococci (Murray, 1997). resistance to fluoroquinolones (ciprofloxacin and levofloxacin) is mediated by alteration of target enzymes, including DNA gyrase and topoisomerase IV, as well as by decreasing intracellular accumulation (Piddock, 1999). Resistance to gentamicin is due to the low permeability of the *E. faecium* cell wall to the large gentamicin molecules, but resistance to high-level gentamicin is conferred by alteration of the binding site in the 30S ribosomal subunit or production of modifying enzymes that lead to inactivation of gentamicin (Osuka et al., 2016). Although both vancomycin and teicoplanin belong to glycopeptide antibiotics, teicoplanin still maintains activity against some VRE-fm isolates in the study. It may be caused by the carrying *vanB* gene, making the isolate lower level resistant to vancomycin and susceptible to teicoplanin (Kuzma et al., 2022).

Because *E. faecium* is becoming increasingly resistant to traditional antibiotics, it is urgent to find new antimicrobial agents as a supplement to traditional antibiotics. One promising agent with antimicrobial activity is unsaturated fatty acids for the prevention and treatment of microbial infections (Chanda et al., 2018). Several studies have shown that unsaturated fatty acids can fight against a wide range of bacteria species (Sun et al., 2016; Churchward et al., 2017; Pushparaj Selvadoss et al., 2018). In the study, six EFAs are used to test antimicrobial activity against VRE-fm. In terms of the chemical structure of EFAs (for example, the number of double bonds, the relative position of the double bonds and the carboxyl group) which was shown in our previous work (Wang et al., 2022), it is difficult to find a pattern to explain the different antibacterial activities displayed by them. The antibacterial effect of EFAs may depend mainly on the interaction between EFAs and the cell membrane of VRE-fm strains, so it is not possible to find a pattern solely from the chemical structure of EFAs. The cell wall of strain V27 adsorbed a large number of DHA molecules was observed by SEM. It suggests that DHA may play an inhibitory role in interfering with the growth by preventing the nutrients transportation into the cell, which is also discovered by Dasa et al. (Dasa et al., 2016) No bacteria with significantly damaged cell membranes were observed, which indicates that direct destruction of the cell membrane of VRE-fm is not the primary antibacterial mechanism of DHA. This result is different from that observed by Sun et al. (Sun et al., 2016) Besides, Cartron et al. found that cis-6-hexadecanoic acid could disrupt the proton motive force and the electron transfer pathways of *Staphylococcus aureus* (Cartron et al., 2014), while Won et al. reported that oleic acid could inhibit the membrane enzymes of *Streptococcus mutans* (Won et al., 2007), which may be also involved in the antibacterial effects of DHA in the current study.

Biofilms play a crucial role in the pathogenesis of *E. faecium* infections, as they protect bacteria from the host’s immune response and improve their resistance to antibiotics. According to the reports by Maurya et al. (Maurya et al., 2021), the maximum biofilm formation of *E. faecium* was achieved in the TSB medium, followed by brain heart infusion broth and Luria Bertani broth. Thus, TSB was selected as the basis in the current study. Since glucose has a significant impact on *E. faecalis* biofilm formation (Tendolkar et al., 2004), different concentrations of glucose were added to TSB to test the biofilm formation of VRE-fm isolates. Finally, TSB+2% glucose medium was selected for the subsequent biofilm study. It is well known that biofilm cells are generally more resistant to antibiotics than their planktonic counterparts, due to the thick mature biofilm, slower metabolic rate, and higher rates of horizontal gene transfer of antibiotic resistance genes within the biofilm community (Komiyama et al., 2016; Lim et al., 2017). Therefore, biofilm-associated *E. faecium* infections are often difficult to eradicate (Ch’ng et al., 2019). Multidrug resistance and biofilm make the treatment of VRE-fm strain infection even more challenging. Numerous studies indicate that unsaturated fatty acids have the activity of inhibiting or eradicating of biofilms formed by a wide range of bacteria, including even fungi (Kim et al., 2018; Ramanathan et al., 2018; Cui et al., 2019; Wang et al., 2022). These findings are similar to those in the current study. It suggests that EFAs may play a role in the clinical treatment of infections in the future.

The initial attachment is the first step in the formation of a biofilm. Various surface adhesins are involved in this stage. The absence of surface adhesins, including enterococcal surface protein (Esp), aggregation substance (Agg), and adhesion to collagen of *E. faecium* (Acm), will reduce adherence to human cells and weaken biofilm formation (Nallapareddy et al., 2007; Soares et al., 2014). Hydrolases, including secreted antigen A (SagA), autolysin (AtlA), and gelatinase (GelE), also play a crucial role in biofilm formation. SagA, which is secreted during biofilm formation, is an important component of the biofilm matrix. It binds to a broad spectrum of extracellular matrix proteins. When SagA is hydrolyzed by proteinase K, the thickness of the biofilm will decrease (Paganelli et al., 2015). autolysin (AtlA) plays a role in the stage of biofilm growth and maturation and is mainly responsible for facilitating the release of eDNA by hydrolyzing *E. faecium* cells (Xie et al., 2022). GelE mainly hydrolyzes gelatin, collagen, casein, and hemoglobin, which assists AtlA in the release of eDNA (Dunny et al., 2014; Kiruthiga et al., 2020). In the study, the *esp*, *sagA*, and *acm* genes were detected in 20/20 (100%), while the *atlA* gene was detected in 19/20 (95.0%) VRE-fm isolates. Only one isolate with low biofilm-forming ability did not detect the *atlA* gene. The results suggest that the *atlA* gene alone cannot determine whether an isolate can form a biofilm but may affect the ability of biofilm formation. However, *agg* and *gelE* genes were not detected in any isolates. This may be because the two genes are present mainly in *E. faecalis* (Kiruthiga et al., 2020), they are not crucial genes for *E. faecium* to form a biofilm.

In terms of eradicating biofilms, in addition to directly killing VRE-fm, further study is needed on how EFAs affect biofilm-related genes which include *acm*, *atlA*, *esp*, and *sagA*. GLA with the highest biofilm eradication rate, and six VRE-fm isolates (V05, V06, V08, V09, V22, and V27) with moderate biofilm formation ability were used to illustrate the mechanism. In this study, GLA could significantly down-regulate the expression of the *atlA* gene in VRE-fm strains, which inhibit the release of eDNA, may result in the eradication of biofilm. The finding suggests that GLA may offer a potential alternative to traditional antibiotics to combat *E. faecium* infections. The *acm* gene was up-regulated in two isolates under the action of GLA, which suggests that these isolates may resist EFAs eradicating or repairing biofilm through high expression of the *acm* gene. These inferences require further work to confirm.

In summary, DHA and GLA showed excellent antibacterial and antibiofilm activity against VRE-fm isolates in the current study, and they are promising next generation antimicrobial agents. Through analysis of relative expression levels of biofilm-related genes, we found that the expression level of *atlA* gene in VRE-fm strains was significantly down-regulated, which may be an important molecular mechanism for EFAs to eradicate biofilms.

## 4 Materials and methods

### 4.1 Strains and culture conditions

A total of 20 clinical VRE-fm isolates (non-replicative) were collected from the Department of Infectious Diseases and Clinical Microbiology, Beijing Chao-Yang Hospital, Capital Medical University (Beijing, China). These strains were identified using matrix-assisted laser desorption/ionization-time of flight (MALDI-TOF) (VITEK-MS; bioMérieux, France; IVD version 3.0). The verified strains were routinely refreshed from frozen stocks at -20°C and inoculated at least twice on Columbia blood agar at 35°C for 24 h before all experiments.

### 4.2 Antimicrobial susceptibility testing

The antimicrobial susceptibility of *E. faecium* to penicillin (P), ampicillin (AM), vancomycin (VA), tigecycline (TGC), ciprofloxacin (CIP), levofloxacin (LEV), tetracycline (TE), quinupristin-dalfopristin (QDA), erythromycin (E), nitrofurantoin (NIT), linezolid (LZD), high-level streptomycin (STR), and high-level gentamicin (GM), were tested by broth microdilution using the Vitek 2 compact system (bioMerieux, Marcy l’Etoile, France). The minimum inhibitory concentration (MIC) of teicoplanin (TEC) was determined by Etest (bioMerieux, Marcy l’Etoile, France). The interpretation of sensitive (S), intermediate sensitive (I), and resistant (R) was carried out according to the guideline of Clinical and Laboratory Standards Institute (CLSI, 2023). Because the CLSI guideline does not offer recommended MIC susceptibility breakpoints for TGC against *E. faecium*, the MICs of TGC were analyzed using the European Committee on Antimicrobial Susceptibility Testing (EUCAST, 2023). *E.* faecalis ATCC 29212 was used as a quality control strain for the antimicrobial susceptibility testing.

### 4.3 Growth inhibition assay

The antimicrobial activity of EFAs (ALA, EPA, DHA, LOA, GLA, and AA) was evaluated using a microdilution method as previously reported (Wang et al., 2022). Briefly, VRE-fm isolates (1.0-2.0 × 10^8^ CFU/mL) inoculated 1:200 into each well of 96-well round bottom microtiter plates with 200 μL of Mueller Hinton II broth containing different concentrations of EFAs. The EFAs were two-fold diluted from 0.125 mM to 1 mM. The MIC values were read and recorded as the lowest concentration of compounds that inhibited visible growth of bacteria after a 24 h incubation period at 35°C. All experiments were performed at least three times.

### 4.4 Scanning electron microscopy

To investigate the effect of EFAs on the morphology of VRE-fm cells, the strain V27 incubated with DHA was selected for SEM imaging. Strain V27 at a final concentration about 7.5×10^5^ CFU/mL was incubated with and without 1 mM DHA for 1 h with shaking at 35°C. The samples were fixed in 2.5% glutaraldehyde for 5 h at 4°C, then were dehydrated in a sequential-graded ethanol (30%, 50%, 70%, 80%, 90%, and 100%), and then two times with 100% ethanol for 15 min. Finally, the samples were sputter coated with gold, followed by microscopic examinations by using a SU8020 SEM (Hitachi, Japan) (Rani et al., 2022).

### 4.5 Biofilm formation assay

For biofilm formation, the method described by Cruz et al. was followed with some modifications (Cruz et al., 2018). Briefly, VRE-fm cultures were diluted 1:100 in fresh trypticase-soy broth (TSB) containing 0.5%, 1% or 2% glucose. Then, 200 µL of suspension (1×10^6^ CFU/mL) was added to each well of the microtiter plate and incubated at 35°C for 24 h. Wells with sterile medium were used as negative controls.

To determine the biofilm mass, the wells were washed three times with phosphate buffered saline (PBS; pH 7.2) to remove planktonic cells. The remaining attached cells were fixed with 200 μL methanol for 15 min, stained with 200 μL crystal violet (0.2%) for 20 min, and washed with distilled water to remove excess crystal violet. Then, the bound crystal violet was extracted with 200 μL of 33% acetic acid. The OD values were measured at 630 nm using a Multiskan EX microplate photometer (Thermo Fisher Scientific, Waltham, MA, United States). The experiments were independently repeated three times. The OD cut-off value (ODc) for biofilm formation was defined as three standard deviations above the mean OD value of the negative control.

The isolates were classified as strong biofilm producers (OD _630 nm_ > 4×ODc), moderate biofilm producers (2×ODc < OD _630 nm_ ≤ 4×ODc), weak biofilm producers (ODc < OD _630 nm_ ≤ 2×ODc), and non-biofilm producers (OD _630 nm_ ≤ ODc) (El-Houssaini et al., 2019).

### 4.6 Biofilm inhibition and eradication assay

For the biofilm inhibition assay, *E. faecium* isolates were inoculated into microtiter plates with TSB + 2% glucose (TSBG) containing 1 mM EFAs. TSBG medium without EFAs was used as control. After incubation of microplates at 35°C for 24 h, the biofilm formed was measured using the crystal violet method as described above. The biofilm inhibition rate (%) was calculated according to the formula: Inhibition (%) = (OD _control_ - OD _sample_)/OD _sample_ × 100% (Wang et al., 2021).

For the biofilm eradication assay, mature biofilms of *E. faecium* were pre-formed in TSBG medium culturing for 24 h. After incubation, the non-adherent cells were removed by washing with PBS twice. Then, TSBG containing 1 mM EFAs was added to the wells. The mature biofilms treated with fresh TSBG medium were used as a control. The microtiter plates were incubated at 35°C for an additional 24 h, and the biofilm mass was detected using the crystal violet method described above. The biofilm eradication rate (%) was calculated according to the formula: Eradication (%) = (OD _control_ - OD _sample_)/OD _sample_ × 100% (Wang et al., 2021).

### 4.7 DNA extraction and detection of biofilm-associated genes

To detect the presence of biofilm-associated genes from *E. faecium* isolates, a qPCR assay was used. Genomic DNA was extracted from strains inoculated on Columbia blood agar using a TIANamp Bacteria DNA kit (Tiangen Biotech (Beijing) Co., Ltd., Beijing, China) according to the manufacturer’s protocol. Specific primers were used for the PCR testing of all *E. faecium* isolates to detect biofilm-related genes encoding Acm, Agg, AtlA, Esp, GelE, and SagA. The primers used for the detection are shown in **Table 4**. The PCR procedure was 95°C for 3 min, 40 cycles (95°C for 15 s, and 60°C for 1 min).

**Table 4:** List of primers used in this study.

Primers	Primer sequence (5′–3′)	Product length (bp)	References
*16S*-F *16S*-R	GCCACATTGGGACTGAGACA TGCCACCTACGTATTACCGC	237	This study(MT729967.1)
*acm*-F *acm*-R	GGCCAGAAACGTAACCGATA AACCAGAAGCTGGCTTTGTC	135	(Rathnayake et al., 2012)
*agg*-F *agg*-R	AGCCAACTATGGCGGAATCA CCTGTGAAACGATCATGGGT	75	This study (U91527.1)
*atlA*-F *atlA*-R	TAGCGGCTTTGATGGCTCTT AGCTTCCGACTTCATCCGTTG	143	This study (CP064343.1)
*esp*-F *esp*-R	GAGCGGAGACACGAATCCAT TTCCCGCTAACTCGTGGATG	104	This study (AF417507.1)
*gelE*-F *gelE*-R	TGGGATGGAAAAGCAATGCG AAACCGGCAGTATGTTCCGT	122	This study (MW922033.1)
*sagA*-F *sagA*-R	AAAGAAGCACGCGAACAAGC GTTGCTGAGCTTTGTGCAGT	74	This study (LN714770.1)

### 4.8 RNA extraction and qRT-PCR

To determine the effects of GLA on the transcription of *E. faecium* genes related to biofilms, the gene expression levels of *acm*, *atlA*, *esp*, and *sagA* were evaluated by a qRT-PCR method. Sequences of gene-specific primers and the reference gene encoding 16S rRNA (*16S*), which serves as an internal control for the determination of gene expression level, are presented in **Table 4**. Some primers used in this study were designed by Primer-BLAST (https://www.ncbi.nlm.nih.gov/tools/primer-blast), according to selected sequences obtained from the GenBank database.

The mature biofilm of *E. faecium* was established according to the previous method. After the biofilm was treated with or without GLA for 24 h, the cells were collected by centrifugation (5000 × g for 10 min). Total RNA from *E. faecium* was extracted using the RNA extraction reagent kit (Tiangen Biotech, Beijing, China) according to the manufacturer′s instructions. The concentration, purity, and quality of the isolated RNA samples were determined using a Nano Drop One Spectrophotometer (Thermo Scientific, Waltham, MA, USA). RNA (1 μg) from each sample was immediately reverse transcribed into cDNA using SuperScript™ III First-Strand Synthesis SuperMix (Invitrogen, Carlsbad, CA, USA). qRT-PCR was analyzed using the PowerUp SYBR Green Master Mix (Applied Biosystems, Life Technologies, Austin, USA) in an Applied Biosystems 7500 real-time PCR system (Applied Biosystems, Waltham, MA, USA). The amplification reactions were carried out in a 20 µL reaction volume involving 10 μL of 2× PowerUp SYBR Green Master Mix, 2 μL of cDNA, 1 μL of Forward Primer, 1 μL of Reverse Primer, and 6 μL of H_2_O. The reaction conditions were 95°C for 3 min, 40 cycles (95°C for 15 s, and 60°C for 1 min). Gene expression was normalized with the reference gene *16S* using 2^−ΔΔCT^ method (Livak and Schmittgen, 2001).

### 4.9 Statistical analysis

Statistical analysis was performed using the IBM SPSS Statistics 20.0 software program (IBM, Armonk, NY, USA). Data were expressed as means ± standard deviation (SD) of at least three independent experiments. Statistical comparison between two groups was performed using Student’s *t*-test. Multiple comparisons between the control and different treatment groups were analyzed using one-way analysis of variance (ANOVA) followed by Dunnett’s t test. A *P*-value < 0.05 was considered statistically significant.

## Data availability statement

The original contributions presented in the study are included in the article/supplementary material. Further inquiries can be directed to the corresponding authors.

## Ethics statement

The studies involving humans were approved by Ethics Committee of Beijing Chao-Yang Hospital, Capital Medical University. The studies were conducted in accordance with the local legislation and institutional requirements. Written informed consent for participation was not required from the participants or the participants’ legal guardians/next of kin in accordance with the national legislation and institutional requirements.

## Author contributions

MW: Conceptualization, Methodology, Project administration, Writing – original draft. PW: Methodology, Writing – original draft. TL: Investigation, Writing – original draft. QW: Validation, Writing – original draft. MS: Formal Analysis, Writing – original draft. LG: Supervision, Writing – review & editing. SW: Funding acquisition, Visualization, Writing – review & editing.
